# Minimally invasive versus open multivisceral resection for rectal cancer clinically invading adjacent organs: a propensity score-matched analysis

**DOI:** 10.1007/s00464-024-10844-5

**Published:** 2024-04-24

**Authors:** Shinya Abe, Hiroaki Nozawa, Kazuhito Sasaki, Koji Murono, Shigenobu Emoto, Yuichiro Yokoyama, Hiroyuki Matsuzaki, Yuzo Nagai, Takahide Shinagawa, Hirofumi Sonoda, Soichiro Ishihara

**Affiliations:** Department of Surgical Oncology, Graduate School of Medicine, The University of Tokyo, 7-3-1 Hongo, Bunkyo-Ku, Tokyo, 113-0033 Japan

**Keywords:** Minimally invasive surgery, Open surgery, Multivisceral resection, Locally advanced rectal cancer, Outcome, Propensity score-matching

## Abstract

**Background:**

Minimally invasive surgery (MIS), such as laparoscopic and robotic surgery for rectal cancer, is performed worldwide. However, limited information is available on the advantages of MIS over open surgery for multivisceral resection for cases clinically invading adjacent organs.

**Patients and methods:**

This was a retrospective propensity score-matching study of consecutive clinical T4b rectal cancer patients who underwent curative intent surgery between 2006 and 2021 at the University of Tokyo Hospital.

**Results:**

Sixty-nine patients who underwent multivisceral resection were analyzed. Thirty-three patients underwent MIS (the MIS group), while 36 underwent open surgery (the open group). Twenty-three patients were matched to each group. Conversion was required in 2 patients who underwent MIS (8.7%). R0 resection was achieved in 87.0% and 91.3% of patients in the MIS and open groups, respectively. The MIS group had significantly less blood loss (170 vs. 1130 mL; *p* < 0.0001), fewer Clavien–Dindo grade ≥ 2 postoperative complications (30.4% vs. 65.2%; *p* = 0.0170), and a shorter postoperative hospital stay (20 vs. 26 days; *p* = 0.0269) than the open group. The 3-year cancer-specific survival rate, relapse-free survival rate, and cumulative incidence of local recurrence were 75.7, 35.9, and 13.9%, respectively, in the MIS group and 84.5, 45.4, and 27.1%, respectively, in the open group, which were not significantly different (*p* = 0.8462, 0.4344, and 0.2976, respectively).

**Conclusion:**

MIS had several short-term advantages over open surgery, such as lower complication rates, faster recovery, and a shorter hospital stay, in rectal cancer patients who underwent multivisceral resection.

**Graphical abstract:**

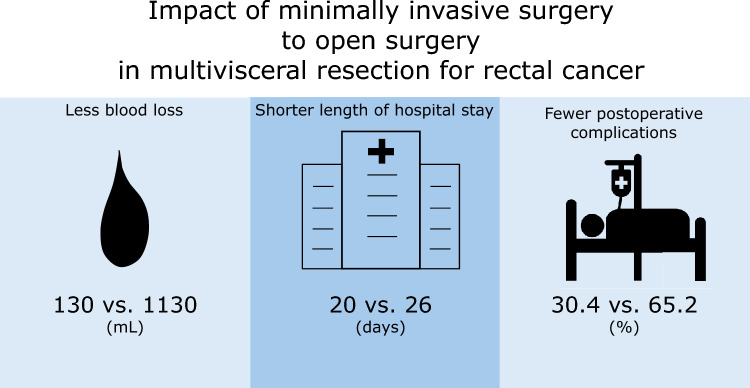

A preoperative multidisciplinary approach, such as chemoradiotherapy, chemotherapy, or both, has been applied to the treatment of advanced rectal cancer. However, complete resection remains the standard treatment for rectal cancer to achieve a good prognosis [1]. R0 resection is a better prognostic factor, even for cT4b tumors suspected to directly invade other organs and structures. Therefore, multivisceral resection is performed to achieve a negative surgical margin in advanced cancers [2, 3].

Since laparoscopic surgery for advanced rectal cancer is a technically demanding procedure, several large randomized controlled trials (RCT) excluded T4 rectal cancer [4–7]. Although two RCTs failed to report that laparoscopy was not inferior to open surgery for successful pathological rectal resection [6, 7], the other two RCTs demonstrated that the oncological outcomes of laparoscopy were not worse than those of open surgery. An additional RCT also showed the advantages of laparoscopy, such as early postoperative recovery and a shorter hospital stay [5]. Only one retrospective study reported the benefits of laparoscopic surgery over open surgery for T4 rectal cancer based on evaluations of short- and long-term outcomes [8], and two retrospective studies demonstrated the utility of robotic surgery for cT4 rectal cancer [9, 10]. Both studies showed a low open surgery conversion rate from 0 to 2.8%. However, these studies on T4 rectal cancer included from 26.9% to 61.1% multivisceral resection, and no studies have evaluated those for rectal cancer treated with multivisceral resection only.

Therefore, the present study examined the short- and long-term outcomes of minimally invasive versus open multivisceral resection for rectal cancer using a propensity score-matched analysis.

## Patients and methods

### Patients

**Fig. 1:**
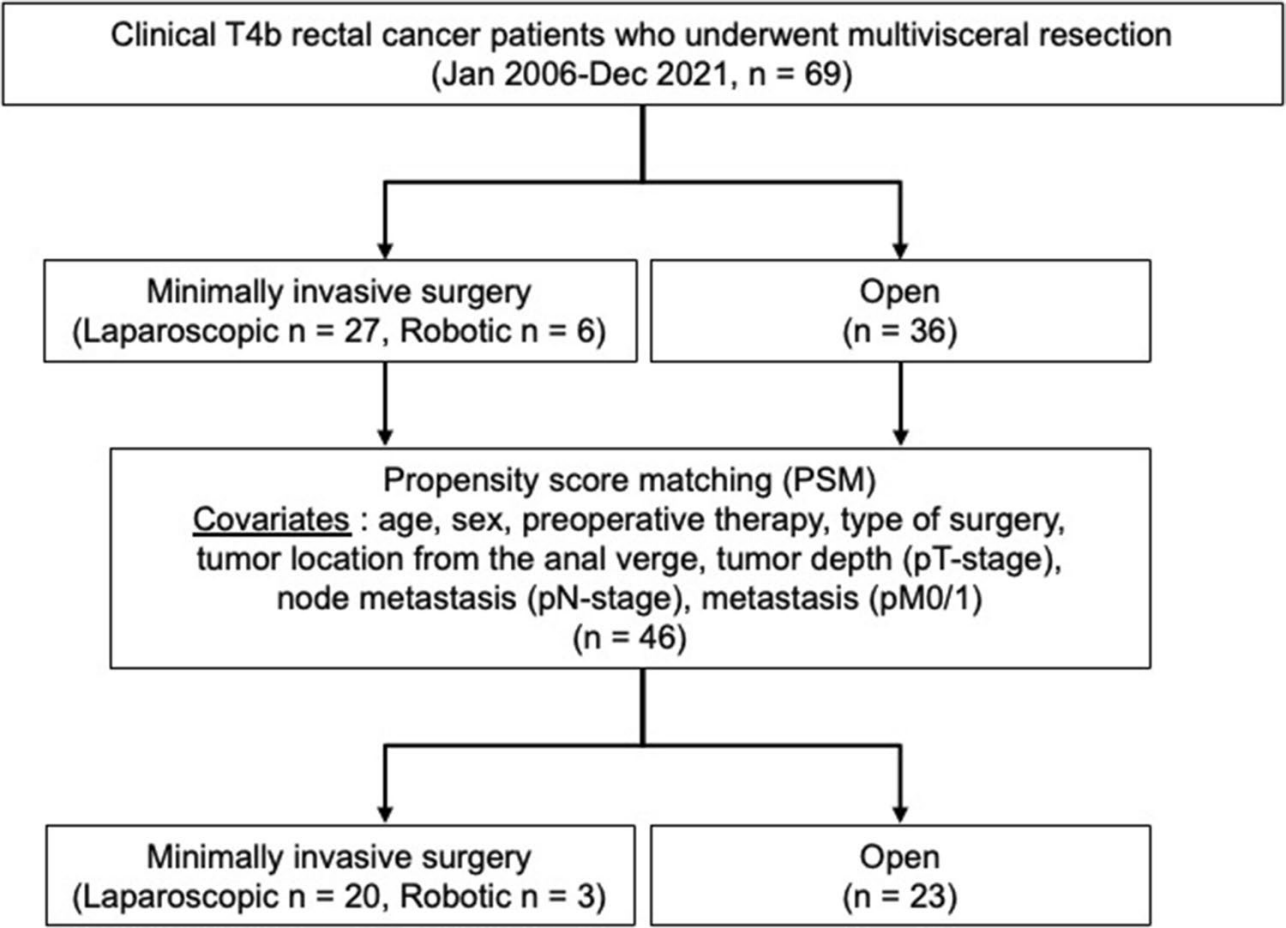
Study cohort selection and propensity score-matching

Between January 2006 and December 2021, 69 consecutive rectal cancer patients who underwent multivisceral resection for curative intent at the University of Tokyo Hospital were retrospectively reviewed (Fig. 1). The present study was approved by the Institutional Ethics Committee of the University of Tokyo (No. 3252-[15]). Informed consent was obtained through an opt-out method due to the study’s retrospective nature.

### Treatment

**Fig. 2:**
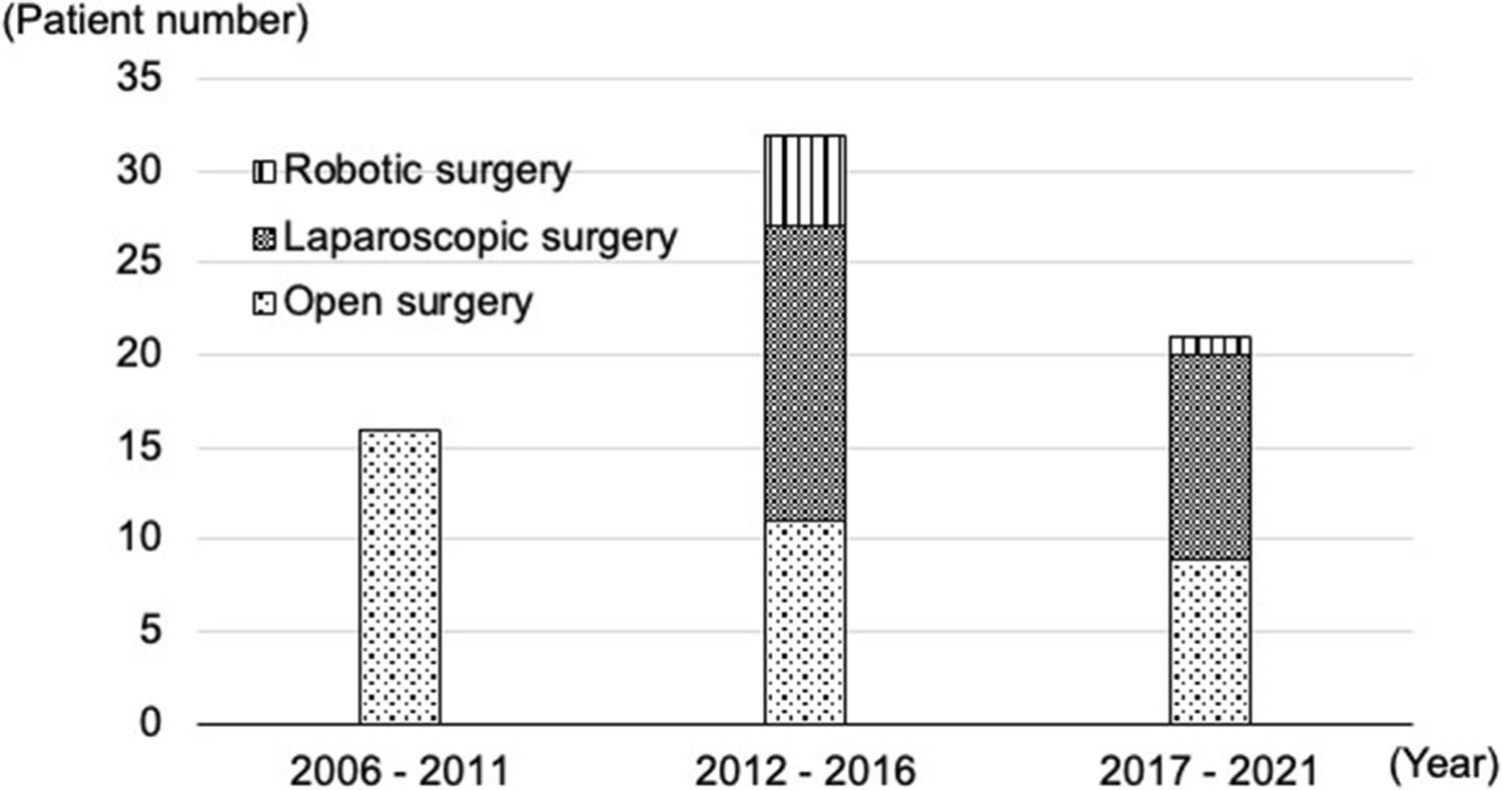
Change in patients who underwent multivisceral rectal resection

Laparoscopic surgery has been selectively applied for cT4b colorectal cancer in our institute since 2012. Robotic surgery also became a treatment option for patients with cT4b rectal cancer in 2013. MIS is currently selected for eligible patients (Fig. 2). At least one surgeon qualified based on the endoscopic surgical skill qualification system of the Japan Society for Endoscopic Surgery participated in each minimally invasive surgery (MIS) as an operator and supervisor [11–13]. Laparoscopic surgery was performed using five ports, while robotic surgery was conducted using da Vinci Si or Xi surgical systems. Conversion from MIS to open surgery was defined as the need to perform a laparotomy before completion of TME. Preoperative chemoradiotherapy was long-course radiation (50.4 Gy in 28 fractions), mainly with a 5-fluorouracil-based oral administration. Some patients received CAPOX or FOLFOX with/without targeting antibodies as preoperative chemotherapy. Lateral pelvic lymph node dissection was selectively performed for patients with lymph nodes of a longitudinal diameter ≥ 8 mm on computed tomography before preoperative therapies [14, 15]. Adjuvant chemotherapy was inconsistent and decided by each patient’s preference and surgeon’s direction because of the relatively long study period.

### Follow-up

Postoperative surveillance was conducted according to the surveillance protocol in the Japanese Society for Cancer of the Colon and Rectum guidelines [16]. Patients were followed by measuring carcinoembryonic antigen levels (every 3 months), performing CT scans (every 6 months), and conducting colonoscopy (every 12 months) for 5 years after surgery.

### Pathological evaluation

All resected specimens were pathologically analyzed after surgery. According to the Japanese Classification of Colorectal, Appendiceal, and Anal Carcinoma of the Japanese Society for Cancer of the Colon and Rectum [17], the TNM classification and stage were assessed. Therefore, patients with pathological regional lymph node-positive numbers from one to three and more than four were classified as (yp)N-stage 1 and 2, respectively. Patients with pathological lateral lymph node-positive cancer were classified as (yp)N-stage 3.

### Statistical analysis

All continuous data were presented as the median and interquartile range (IQR). Propensity score-matching was performed using eight factors: sex, age, preoperative treatment, type of surgery, (yp)T-stage, (yp)N-stage, (yp)M-stage, and tumor location from the anal verge. We performed 1:1 matching between the MIS and open groups using nearest neighbor matching (caliper = 0.2) of the standard deviation of the propensity score logit. The significance of differences was analyzed using the chi-square test for categorical variables or the Mann–Whitney U test for continuous variables. Cancer-specific survival (CSS) was defined as the interval between the date of rectal surgery and disease-specific death or the last follow-up. Relapse-free survival (RFS) was defined as the interval between the date of rectal surgery and recurrence, the last follow-up, or death. The Kaplan–Meier method with the Log-rank test was used to estimate and compare patient survival. All analyses were performed using JMP Pro 15.0 software (SAS Institute Inc., Cary, NC, USA). *p* values < 0.05 were considered to be significant.

## Results

### Clinical characteristics

Among 69 patients, 33 underwent MIS (the MIS group, including six patients who underwent robotic surgery), while 36 underwent open surgery (open group) (Fig. 1). Table 1 shows patient and tumor characteristics as well as perioperative therapy for the entire cohort and matched cases. Before matching, the surgical procedure significantly differed; intersphincteric resection and total pelvic exenteration were only performed in the MIS and open groups, respectively. Additionally, colostomy was more frequently performed before rectal surgery in the open group. After matching, patients who underwent low anterior resection, abdominal perineal resection, and high anterior resection were included in both groups. No significant differences were observed in adjuvant chemotherapy or tumor factors, including the status of resection margin. Among 15 patients (32.6%) who did not receive preoperative treatment or adjuvant chemotherapy, eight, six, and one were in pStages II, III, and IV, respectively. Six of the patients in pStage II were pT4b. The primary reason was an advanced age; four and two patients in Stages II and III were older than 75 years old.

**Table 1:** Association between baseline characteristics before and after propensity score-matching

Variables	Overall cohort			Matched cohort		
	MIS	Open		MIS	Open	
	(*n* = 33)	(*n* = 36)	*p* value	(*n* = 23)	(*n* = 23)	*p* value
Patient factors						
Age (years)*	67 (57–74)	63 (53–75)	0.4598	67 (51–76)	63 (52–76)	0.7751
Gender						
Male	19 (57.6)	17 (47.2)	0.3893	13 (56.5)	9 (39.1)	0.2365
Female	14 (42.4)	19 (51.8)		10 (43.5)	14 (60.9)	
BMI (kg/m^2^)*	21.0 (19.0–23.7)	20.6 (19.3–22.2)	0.7548	21.3 (19.3–23.5)	20.3 (19.0–21.5)	0.3545
ASA-PS score						
I	12 (36.4)	11 (30.6)	0.7936	7 (30.4)	8 (34.8)	0.9507
II	20 (60.6)	23 (63.9)		15 (65.2)	14 (60.8)	
III	1 (3.0)	2 (5.5)		1 (4.4)	1 (4.4)	
Charlson comorbidity index						
0	25 (75.8)	32 (88.8)	0.3516	17 (73.9)	21 (91.2)	0.2607
1	4 (12.1)	2 (10.6)		4 (17.4)	1 (4.4)	
2	4 (12.1)	2 (10.6)		2 (8.7)	1 (4.4)	
Perioperative therapy factors						
Preoperative therapy						
Chemoradiotherapy	11 (33.3)	15 (41.7)	0.5150	7 (30.4)	8 (34.8)	0.9054
Radiotherapy	1 (3.0)	1 (2.8)		1 (4.4)	1 (4.4)	
Chemotherapy	2 (6.1)	5 (13.9)		1 (4.4)	2 (8.7)	
None	19 (57.6%)	15 (41.7)		14 (60.8)	12 (52.1)	
Preoperative decompression						
Stent	1 (3.0)	1 (2.8)	0.0787	1 (4.4)	0	0.0558
Colostomy	1 (3.0)	7 (19.4)		1 (4.4)	6 (26.1)	
None	31 (94.0)	28 (77.8)		21 (91.2)	17 (73.9)	
Adjuvant chemotherapy	15 (45.5)	12 (33.3)	0.3024	10 (43.5)	6 (26.1)	0.2138
Tumor factors						
Distance from AV (cm)*	6.0 (2.3–10)	7.5 (3.9–12)	0.2839	9.0 (3.0–11)	8.0 (6.0–12)	0.7327
Residual tumor size (cm)*	6.0 (3.9–7.1)	5.0 (3.0–6.9)	0.3934	6.0 (4.0–7.7)	5.2 (3.4–7.5)	0.3791
(y)p T-stage						
T2	2 (6.0)	0	0.3610	0	0	1.0000
T3	9 (27.3)	11 (30.6)		7 (30.4)	7 (30.4)	
T4a	5 (15.2)	7 (19.4)		4 (17.4)	4 (17.4)	
T4b	17 (51.5)	18 (50.0)		12 (52.2)	12 (52.2)	
(y)pN metastasis						
0	14 (42.4)	21 (58.3)	0.3120	11 (47.8)	13 (56.5)	0.5134
1	10 (30.3)	9 (25.0)		6 (26.1)	6 (26.1)	
2	6 (18.2)	2 (5.6)		4 (17.4)	1 (4.4)	
3	3 (9.1)	4 (11.1)		2 (8.7)	3 (13.0)	
(y)pM metastasis	7 (21.2)	8 (22.2)	0.9190	5 (21.7)	4 (17.4)	0.7099
Histopathological type						
Differentiated (Well/Moderate)	28 (84.9)	32 (88.9)	0.6187	20 (87.0)	21 (91.3)	0.6347
Others	5 (15.1)	4 (11.1)		3 (13.0)	2 (8.7)	
Resection margin status						
R0	29 (87.9)	34 (94.4)	0.3306	20 (87.0)	21 (91.3)	0.6347
R1	4 (12.1)	2 (5.6)		3 (13.0)	2 (8.7)	
R2	0	0		0	0	
Surgical factors						
Surgical procedure						
Low anterior resection	19 (57.6)	19 (52.7)	0.0008	15 (65.2)	17 (73.9)	0.7945
Abdominal perineal resection	10 (30.3)	5 (13.9)		7 (30.4)	5 (21.7)	
Intersphincteric resection	3 (9.1)	0		0	0	
Total pelvic exenteration	0	6 (16.7)		0	0	
Hartmann’s procedure	0	5 (13.9)		0	0	
High anterior resection	1 (3.0)	1 (2.8)		1 (4.4)	1 (4.4)	
LPN dissection	7 (21.2)	6 (16.7)	0.6297	5 (21.7)	4 (17.4)	0.7099
Harvested lymph nodes*	22 (13–30)	23 (12–38)	0.6522	22 (10–30)	24 (13–35)	0.7835

Values in parentheses are percentages, unless indicated otherwise

*MIS* Minimally invasive surgery, *BMI* Body mass index, *ASA-PS score* American Society of Anesthesiologists-Physical Status score, *AV* Anal verge, *LPN* Lateral pelvic node

*Values are median (interquartile range)

### Characteristics of resected organs and structures

The invasion of adjacent organs and structures before and after propensity score-matching is shown in Table 2. Before matching, resected organs and structures differed in both groups. The seminal vesicle and abdominal wall tended to select for MIS and the bladder, uterus, and prostate gland for open surgery. The proportions of the cases with pathologically confirmed direct invasion to organs or structures, namely those of pT4b among sT4b cases in the MIS and open groups, were 50% (19 of 38) and 30.2% (16 of 53), respectively. After matching, the pT4b-to-sT4b ratios for the abdominal wall and uterus were still clearly different between the groups. The pT4b-to-sT4b ratio in the MIS and open groups were 50% (13 of 26) and 37.9% (11 of 29), respectively.

**Table 2:** Invasion of adjacent organs and structures before and after propensity score-matching

	Overall cohort		Matched cohort	
	MIS	Open	MIS	Open
	(*n* = 33)	(*n* = 36)	(*n* = 23)	(*n* = 23)
sT4b/pT4b				
Bladder	1/1	11/3	1/1	3/0
Vagina	8/6	8/4	4/4	6/3
Uterus	1/1	10/5	1/1	9/5
Ovary	4/1	8/0	4/1	4/0
Seminal vesicle	9/6	3/1	3/3	1/1
Abdominal wall	9/2	1/0	8/1	0/0
Omentum	1/0	0/0	1/0	0/0
Small intestine	1/1	2/1	1/1	1/1
Colon and Rectum	1/1	1/0	1/1	1/0
Prostate grand	3/0	8/2	2/0	3/1
Urinary duct	0/0	1/0	0/0	1/0

*MIS* Minimally invasive surgery

### Short-term outcomes

Table 3 shows operative and postoperative outcomes before and after propensity score-matching. After matching, estimated blood loss was significantly less in the MIS group than in the open group. There were two cases of conversion to open surgery in the MIS group. The reasons for conversion were invasion to the left ovary in one, which was resected under laparotomy by gynecologists. In the other case, invasion of the bladder with the formation of a pelvic abscess caused penetration close to the bladder due to rectal cancer. The rates of postoperative complications of all grades, CD grade ≥ 2 and CD grade ≥ 3, were significantly lower in the MIS group (30.4, 30.4, and 4.4%, respectively) than in the open group (65.2, 65.2, and 26.1%, respectively). Regarding CD grade ≥ 2 postoperative complications, the incidence of any infectious complications was significantly lower in the MIS group than in the open group. Among the patients who developed CD grade ≥ 3 postoperative complications, five were treated with interventions that did not require general anesthesia, namely, CD grade 3a, and two received interventions that were performed under general anesthesia, namely, CD grade 3b. The rate of reoperation within 30 days after surgery did not significantly differ between the two groups, and no mortality was observed in either group. The time to first flatus and length of postoperative stays were significantly shorter in the MIS group than in the open group.

**Table 3:** Operative and postoperative outcomes before and after propensity score-matching

Variables	Overall cohort			Matched cohort		
	MIS	Open		MIS	Open	
	(*n* = 33)	(*n* = 36)	*p* value	(*n* = 23)	(*n* = 23)	*p* value
Operative time (min)*	388 (317–516)	437 (292–758)	0.2665	387 (288–509)	397 (262–584)	0.5028
Estimated blood loss (mL)*	180 (23–508)	1380 (713–2060)	< 0.001	170 (20–510)	1130 (590–2030)	< 0.0001
Conversion to open surgery	2 (6.1)	–	–	2 (8.7)	–	–
Postoperative complication (All grade)	12 (36.4)	21 (58.3)	0.0668	7 (30.4)	15 (65.2)	0.0170
Postoperative complication (CD grade ≥ 2)						
All	11 (33.3)	21 (58.3)	0.0364	7 (30.4)	15 (65.2)	0.0170
Any infection	7 (21.2)	17 (47.2)	0.0218	5 (21.7)	12 (52.2)	0.0306
Surgical site infection (superficial)	0 (0)	1 (2.8)	0.2516	0 (0)	1 (4.4)	0.2353
Surgical site infection (Deep/Organ)	3 (9.1)	9 (25.0)	0.0751	3 (13.0)	7 (30.4)	0.1482
Anastomotic leakage	1 (3.0)	1 (2.8)	0.9502	1 (4.4)	1 (4.4)	1.0000
Urinary infection	2 (6.1)	8 (22.2)	0.0490	1 (4.4)	4 (17.4)	0.1428
Intestinal obstruction/ileus	2 (6.1)	1 (2.8)	0.5014	2 (8.7)	1 (4.4)	0.5468
Urination disorder	2 (6.1)	1 (2.8)	0.5014	1 (4.4)	1 (4.4)	1.0000
Others	1 (3.0)	4 (11.1)	0.1801	1 (4.4)	2 (8.7)	0.5468
Postoperative complications (CD grade ≥ 3)	2 (6.1)	8 (22.2)	0.0490	1 (4.4)	6 (26.1)	0.0319
Reoperation within 30 days after surgery	1 (3.0)	2 (5.6)	0.6034	1 (4.4)	1 (4.4)	1.0000
Mortality	0 (0)	0 (0)	–	0 (0)	0 (0)	–
Time to first flatus (days)*	2 (1–3)	2 (1–3)	0.0214	2 (1–2)	4 (1–4)	0.0307
Time to first oral intake (days)*	7 (5–8)	7 (6–9)	0.1541	6 (5–8)	8 (6–9)	0.2082
Length of postoperative stay (days)*	20 (17–23)	26 (20–35)	0.0036	20 (17–23)	26 (19–37)	0.0269

Values in parentheses are percentages, unless indicated otherwise

*MIS* Minimally invasive surgery, *CD grade*, Clavien–Dindo classification grade

*Values are median (interquartile range)

### Long-term outcomes

**Fig. 3:**
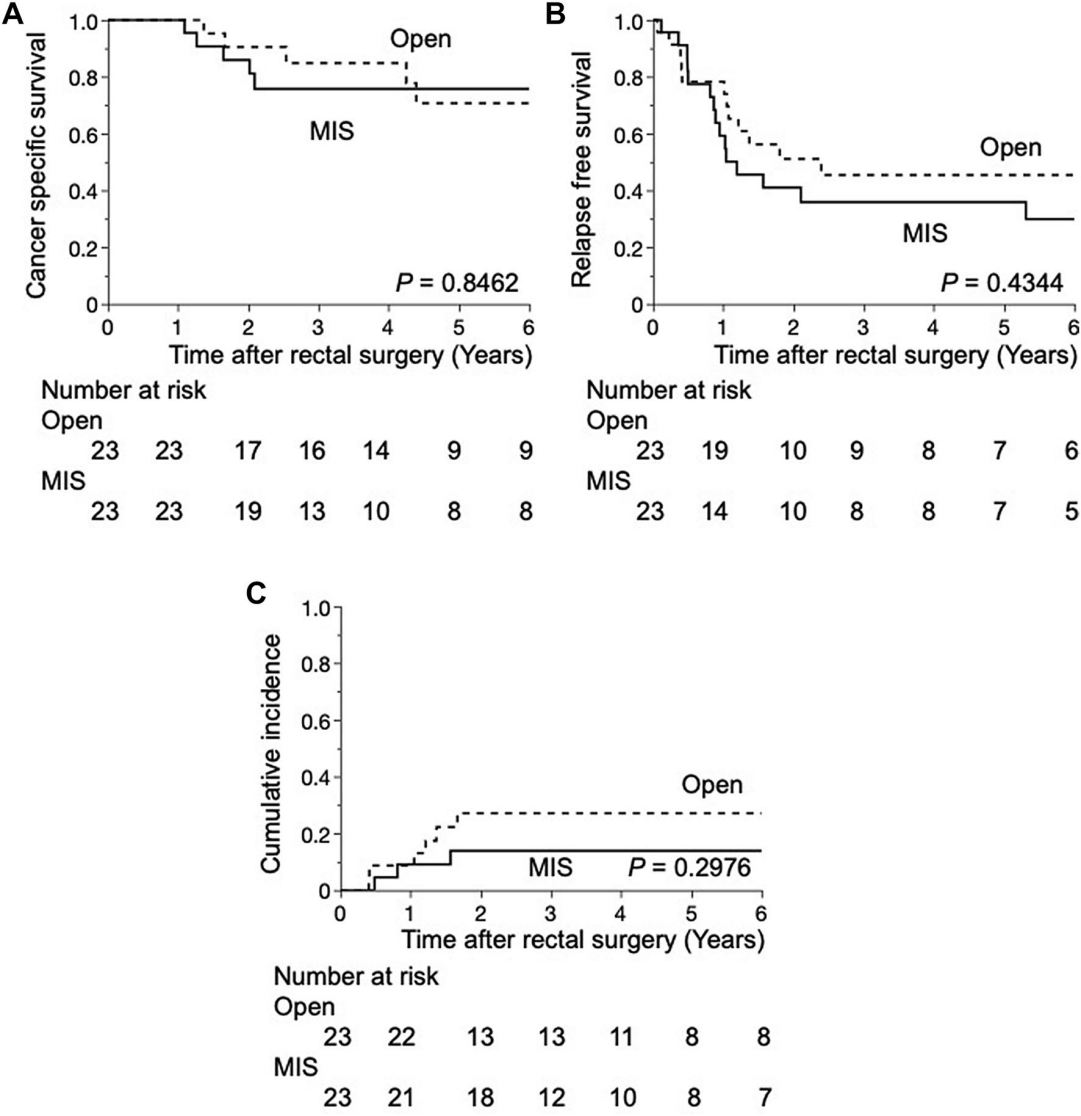
**A** Cancer-specific survival curves. A Kaplan–Meier curve for comparisons between minimally invasive and open multivisceral resection for rectal cancer in propensity-matched pairs (*n* = 46). **B** Relapse-free survival curves. A Kaplan–Meier curve for comparisons between minimally invasive and open multivisceral resection for rectal cancer in propensity-matched pairs (*n* = 46). **C** Cumulative incidence of local recurrence. A Kaplan–Meier curve for comparisons between minimally invasive and open multivisceral resection for rectal cancer in propensity-matched pairs (*n* = 46)

The median follow-up period for matched patients was 3.41 years (IQR 1.90–7.02). Three-year cancer-specific survival rates were 75.7 and 84.5% in the MIS and open groups, respectively (*p* = 0.8462) (Fig. 3A). Three-year RFS rates were 35.9 and 45.4% in the MIS and open groups, respectively (*p* = 0.4344) (Fig. 3B). Regarding local recurrence, the 3-year cumulative incidences of local recurrence were 13.9 and 27.1% in the MIS and open groups, respectively (*p* = 0.2976) (Fig. 3C). The most common site of recurrence was the lung followed by distant lymph nodes in both groups (Table 4).

**Table 4:** Oncological outcomes before and after propensity score-matching

		Overall cohort		Matched cohort	
		MIS	Open	MIS	Open
		(*n* = 33)	(*n* = 36)	(*n* = 23)	(*n* = 23)
Recurrence		19 (57.6)	19 (52.8)	14 (60.9)	12 (52.2)
Multiple		4 (12.1)	5 (13.9)	2 (8.7)	2 (8.7)
Distant*		17 (51.5)	15 (41.7)	12 (52.2)	7 (30.4)
	Lung*	8 (24.2)	7 (19.4)	5 (21.7)	4 (17.4)
	Liver*	5 (15.2)	3 (8.3)	3 (13.0)	3 (13.0)
	Distant lymph node*	6 (18.2)	7 (19.4)	5 (21.7)	4 (17.4)
	Peritoneal*	1 (3.0)	3 (8.3)	1 (4.3)	0
	Bone*	0	1 (2.8)	0	0
	Spleen*	0	1 (2.8)	0	0
Local*		2 (6.1)	6 (16.7)	2 (8.7)	5 (21.7)

Values in parentheses are percentages, unless indicated otherwise

*MIS* Minimally invasive surgery

*Contains duplicates

## Discussion

Previous studies reported the safety and feasibility of MIS for advanced cT4 and pT4 rectal cancer; however, patients who did not undergo multivisceral resection were included in these cohorts [8–10]. Therefore, only rectal cancer patients who underwent multivisceral resection for curative intent were included in the present study. This is the first study to examine the short- and long-term outcomes of multivisceral resection by comparing minimally invasive and open surgeries using propensity score-matching. Regarding short-term outcomes, MIS had significant advantages over open surgery, such as less estimated blood loss, fewer postoperative complications, primarily associated infections, and a shorter time to first flatus and length of postoperative stay. Previous studies confirmed the better short-term outcomes of laparoscopic multivisceral resection over open surgery for advanced colorectal cancer, which is consistent with the present results [18–20].

The longer operative time of MIS than open surgery is a well-known disadvantage. However, the present results showed no significant differences between the groups before and after matching due to the different proportions of invaded adjacent organs and structures.

MIS for T4b colorectal cancer is considered to be technically demanding and is associated with high open surgery conversion rates. In studies that compared laparoscopic to open surgery for colorectal and colon cancers, the conversion rate ranged between 4.9 and 28.2% [19–22]. Regarding rectal cancer, the technical disadvantage was more than colon cancer, and the conversion rate to open surgery was 21.2% [8]. However, the conversion rate was 6.1% in the present study, which is consistent to values reported in recent studies on colorectal cancer. These variations may be due to selection criteria for the surgical approach, improvements in preoperative imaging, differences in surgeons’ experience, and robotic surgery. Two recent retrospective studies on robotic surgery for T4b rectal cancer reported very low conversion rates [9, 10]. Robotic surgery for rectal cancer has a number of advantages, such as enhanced visualization, dexterity, and ergonomics [23]. In the present study, total pelvic exenteration was performed by open surgery. A recent study reported the advantages of MIS for total pelvic exenteration, such as less blood loss and fewer abdominal wound infections [24]. R0 resection was also achieved in 90% of patients; however, the authors also proposed that enough experiences of standard total mesorectal excision (TME) were required before attempting anything beyond TME operations. Therefore, surgical approaches need to be considered based on surgeons’ experiences.

Pathological invasion rates in laparoscopic and open multivisceral resection for colorectal cancer were previously reported to range between 21.2 and 61.5% [8, 19, 21, 25] and between 32.7 and 70.0% [8, 19, 21], respectively. In the present study, pathological invasion rates with MIS and open surgery before matching were 51.5 and 50.0%, respectively, which are consistent with these values. Although half of the patients who underwent multivisceral resection had peritumoral adhesions due to inflammatory reactions, it is essential to resect adhering or invading adjacent organs or structures *en bloc* with clinically T4b tumors in order to achieve R0 resection. Successful R0 resection affects the oncological outcomes of multivisceral resection for colorectal cancer, and has been identified as an independent favorable factor for better OS in both pT4 colon and rectal cancers [8, 21]. We also previously reported that R0 resection resulted in significantly better OS in patients who underwent multivisceral colorectal resection after preoperative treatment [26]. Although R0 resection rates in the MIS and open groups were similar in the present study, when the risk of positive margins is suspected, conversion to open surgery must be considered because MIS demanded technical difficulty due to the lack of tactile sense in MIS.

Regarding long-term outcomes after matching, CSS and RFS were similar between the MIS and open groups during the 3.41-year median follow-up period in the present study, which is consistent with previous findings on T4 rectal cancer [8]. In comparison of recurrence patterns after matching, the local recurrence rate, affected by the quality of surgical procedures, was lower in the MIS group than in the open group, while the distant recurrence rate was higher in the MIS group. Several factors may have contributed to the high rate of distant recurrence in the MIS group. Although we attempted to balance patient backgrounds using propensity score-matching, the percentage of the (y)pN2 stage was slightly higher in the MIS group than in the open group. Furthermore, it was unclear whether adjuvant chemotherapy was effective because most cases of recurrence were observed within 1 year. The characteristics of pT4 tumors generally correlate with increased lymph node metastasis and synchronous distant metastasis. Additionally, pT4 is a risk factor for recurrence in Stage II colorectal cancer. Therefore, adjuvant chemotherapy may have been selected for the majority of patients in our cohort to prevent distant metastasis after complete surgical resection. In the present study, 43.5 and 26.1% of patients in the MIS and open groups received adjuvant chemotherapy, respectively. Although these percentages were unsatisfactory, they were higher in the MIS group than in the open group. These results suggest that minimally invasive treatment facilitates the implementation of adjuvant chemotherapy due to faster recovery after surgery. A meta-analysis of several Japanese randomized controlled trials demonstrated the significant advantage of adjuvant chemotherapy over surgery alone in terms of overall survival and disease-free survival for rectal cancer [27]. Further large-scale studies are warranted to evaluate the effects of adjuvant chemotherapy in patients with locally advanced rectal cancer.

Several limitations need to be addressed. This was a retrospective analysis of a small cohort study in a single institution. Furthermore, the selection of the surgical procedure, MIS or open surgery, was decided by a multidisciplinary review, was not based on a randomized study, and was affected by historical background. Finally, due to a small cohort, invaded organs and structures were not used in propensity score-matching.

## Conclusion

This propensity score-matched study is the first to show the safety and feasibility of MIS in rectal cancer patients who underwent multivisceral resection. The results suggested that MIS had several short-term advantages for the selected patients, such as lower complication rates, faster recovery, and a shorter hospital stay.
